# Game‐related behaviors among children and adolescents after school closure during the COVID‐19 pandemic: A cross‐sectional study

**DOI:** 10.1002/pcn5.37

**Published:** 2022-08-29

**Authors:** Naoki Yamamoto, Yoshiro Morimoto, Hirohisa Kinoshita, Hirokazu Kumazaki, Sumihisa Honda, Ryoichiro Iwanaga, Akira Imamura, Hiroki Ozawa

**Affiliations:** ^1^ Department of Neuropsychiatry, Unit of Translational Medicine Nagasaki University Graduate School of Biomedical Sciences Nagasaki Japan; ^2^ Child and Adolescent Psychiatry Community Partnership Center Nagasaki University Hospital Nagasaki Japan; ^3^ Unit of Medical Science Nagasaki University Graduate School of Biomedical Sciences Nagasaki Japan; ^4^ Center for Child Mental Health Care and Education Nagasaki University Nagasaki Japan; ^5^ Department of Psychiatric Rehabilitation Science Nagasaki University Graduate School of Biomedical Sciences Nagasaki Japan

**Keywords:** addictive behavior, anxiety, COVID‐19, gaming disorder, ICD‐11

## Abstract

**Aim:**

Increased exposure to digital gaming content among youth in recent years has raised serious health concerns. Social restrictions such as school closures, imposed worldwide because of the ongoing COVID‐19 pandemic, may increase exposure to gaming and lead to addictive gaming behavior in young people. In this study, we investigated gaming behaviors among Japanese students during COVID‐19 school closures.

**Methods:**

Students completed questionnaires regarding their living conditions, game‐related behaviors, diagnosis of Internet addiction, psychological difficulties, and the impact of the COVID‐19 pandemic. We compared differences between the responses of potentially at risk for gaming disorder (potentially at risk for gaming disorder; defined in this paper with reference to the ICD‐11 MMS criteria for gaming disorder [PGD]) students who met the criteria for a diagnosis of gaming disorder in ICD‐11 MMS and those of control students. Logistic regression analysis was performed to predict the extent of factors contributing to potential gaming disorder.

**Results:**

Four thousand and forty‐eight participants completed the survey. Compared with control students (93%), potentially at risk for gaming disorder (defined in this paper with reference to the ICD‐11 MMS criteria for gaming disorder, PGD) students (7%) reported playing games for longer times, spending more money on in‐game purchases, were of younger age at the start of game playing, showed a tendency toward Internet dependence, practised school avoidance or absenteeism, and demonstrated the need for psychological support. Moreover, participants in the PGD group reported more anxiety about COVID‐19 than control participants, as well as an increase in game‐playing time and amount of money spent on games during the COVID‐19 pandemic.

**Conclusion:**

These results indicate that young people classified as having a gaming disorder not only exhibit characteristic game‐related behaviors but may be psychologically and socially vulnerable and need special support, especially during the ongoing COVID‐19 pandemic.

## Graphical abstract

- Internet gaming disorder is an increasing concern as children's exposure to smart devices increases.

- Our study set out to investigate changes in young people's game‐related behaviors during the COVID‐19 pandemic school closures in Japan.

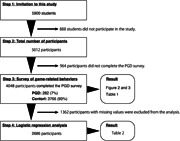

## INTRODUCTION

Devices with easy access to the Internet, such as smartphones and mobile computers, have become commonplace and high‐speed telecommunication infrastructure has expanded. As a result, there is growing concern that children may become immersed in the Internet and digital games, resulting in impaired social functioning.^1^ The volume of digital content targeting adolescents and young adults has increased rapidly, for example the Apple App Store uploads approximately 1000 new apps each day.^2^ Among digital content, games in particular have been suggested to encourage addictive behaviors because of their intermittent reward systems.^3^ Adolescent gamers have been reported to have more sleep disturbances, poorer school performance, more family conflicts, and more emotional, behavioral, and cognitive problems,^4^ suggesting that gaming immersion and resulting health problems are an important public health issue.^5^

The Diagnostic and Statistical Manual of Mental Disorders Fifth Edition (DSM‐5), published in 2013, does not adopt Internet gaming disorder (IGD) as defined by DSM‐5 as a diagnostic category but describes it as a disorder that requires further research.^6^ Subsequently, the 11th revision of the International Classification of Diseases Coding Tool Mortality and Morbidity Statistics (ICD‐11 MMS), released in 2018, adopted gaming disorder as a diagnostic category.^7^ However, there are concerns about the ICD‐11 diagnosis that it is premature to incorporate gaming disorder into a diagnostic manual for mental illness.^8^, ^9^, ^10^ Several researchers have argued that excessive gaming does not necessarily indicate an addictive disorder, that is, gaming may be a coping strategy for stress or a secondary symptom of another mental disorder, obscuring the clinical benefit of labeling a patient with a gaming disorder.^10^, ^11^, ^12^ It has also been suggested that the overpathologization of gaming disorder may exacerbate moral panic about gaming^13^, ^14^, ^15^ and hinder the development of effective, evidence‐based social interventions.^10^ These remarks point to the need for further empirical studies on dysfunction, course, and prognosis, as well as the stability and predictability of the criteria to verify the validity and usefulness of the diagnostic criteria for IGD/gaming disorder.^16^

As of March 31, 2022, the global pandemic of COVID‐19 shows no signs of abating, with a cumulative total of 485,243,022 confirmed infections and 6,137,553 deaths.^17^ To prevent spread of the disease, precautionary measures have been taken, such as wearing masks, physical distancing, and quarantine. Additionally, stress‐relieving measures such as participation in sports, music events, and religious gatherings have become impractical, leading to adverse psychological reactions, such as depression and anxiety.^18^ In such stressful situations where daily activities are restricted, people may turn to substances (such as alcohol) and compensatory behaviors (such as online gaming) to cope with negative emotions.^19^, ^20^ In fact, during the week of March 8–15, 2020, when many state governments in the United States adopted pandemic prevention measures, gaming web traffic in the United States increased by 75%. In March 2020, when a strict lockdown was implemented in Europe, mobile game downloads increased by 19%, the highest download volume ever recorded.^21^, ^22^ Furthermore, there is growing empirical evidence that restrictions on social interactions among youth during the pandemic, as typified by school closures, induced harmful psychological stress and compensatory behaviors in children and adolescents,^23^, ^24^, ^25^, ^26^ making the protection of children and adolescents' mental health during the COVID‐19 pandemic an important public health imperative.

In Japan, nationwide temporary closure of elementary, junior high, and high schools occurred from March 2020, and classes were not held until May of that year. This was the first time in Japan that schools had closed for an extended period because of large‐scale spread of an infectious disease, and the health and educational impact of this closure on students remains unclear. Students could no longer engage in many hobbies or sports, which led to increased opportunities to play online games and raised the risk of gaming disorder.^27^ However, there is still limited information regarding the effects of school closure during the pandemic on children's game‐related behaviors.

Here, we report the results of a cross‐sectional study among Japanese elementary, junior high, and high school students regarding their attitudes and behavioral tendencies toward gaming immediately after school closures as a result of COVID‐19, as well as the impact of the pandemic on these attitudes and behavioral tendencies toward gaming.

## METHODS

### Participants

**Figure 1:**
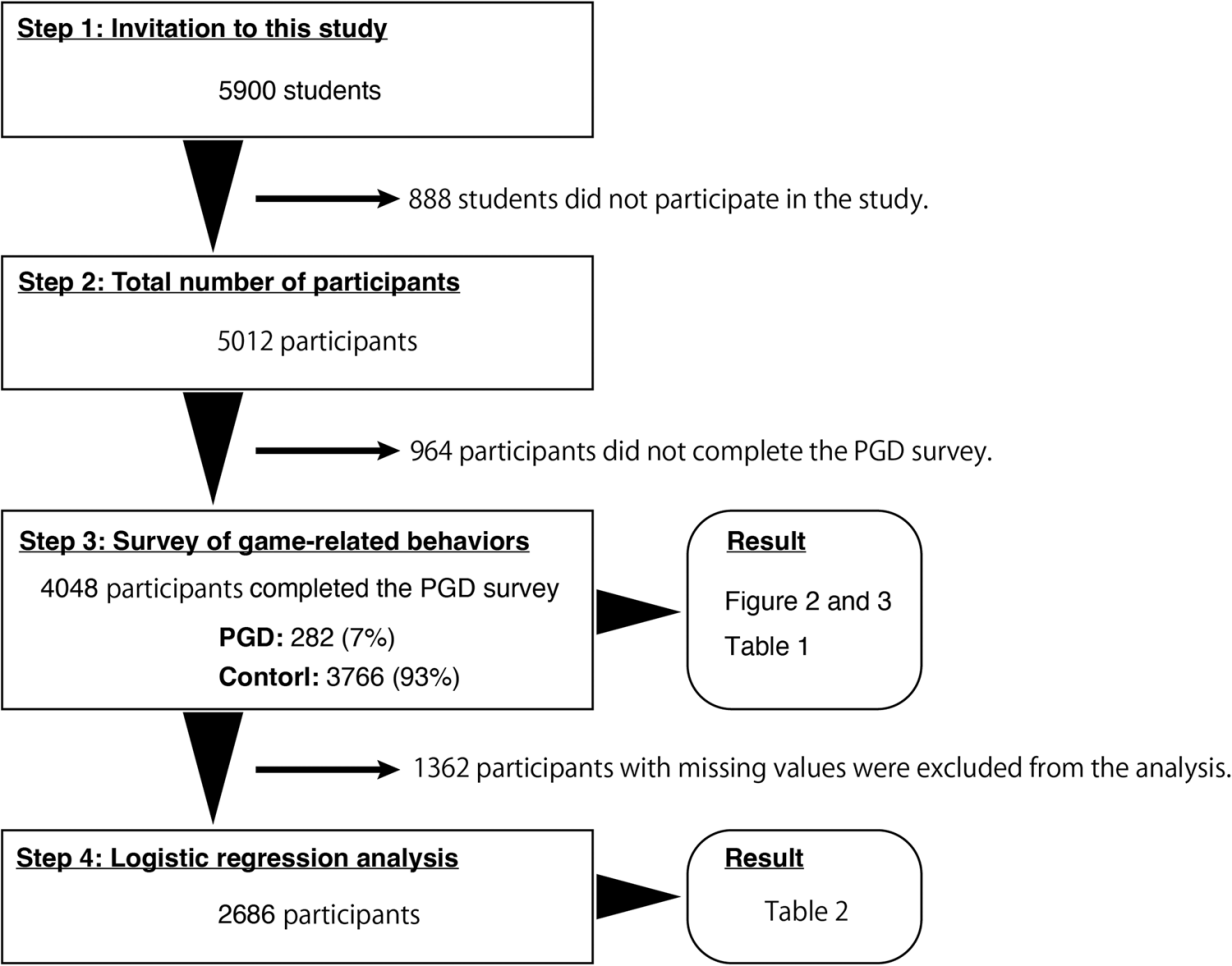
Graphical outline of this study. The study was conducted in four steps: Step (1) 5900 students were invited to participate in the study; Step (2) 5012 participants expressed their willingness to participate in the study; Step (3) 4048 participants completed the PGD survey, with 7% meeting the criteria for PGD, game‐related behaviors and psychosocial vulnerabilities of the PGD and control groups were compared; and Step (4) logistic regression analyses were conducted on data from 2686 participants, not including missing values. PGD, potential gaming disorder.

All experimental procedures were approved by the Ethical Review Committee of Nagasaki University and the study followed the Ethical Guidelines for Medical and Health Research Involving Human Subjects. In December 2020, 5900 students from the fourth grade of elementary school to the third grade of high school (ages 10–18 years) in Nagasaki Prefecture, Japan, were invited to participate in the study (see Figure 1). Informed consent forms were given to both parents and students, and all participants provided written informed consent and voluntarily agreed to participate. The questionnaires were completed at school, and the total number of respondents was 5012, a response rate of 84.9% (see Figure 1). The students were grouped by grade, as shown in Supporting Information Table 1. Students in their third year of junior high school and third year of high school were reluctant to participate because these are important grades for higher education and employment in the Japanese school system.

### Questionnaire and survey items

The survey was conducted at schools after the school closure had ended and school had reopened, using a questionnaire created to survey participants' game‐related behaviors and living environments. To ensure anonymity and validity of the responses, the questionnaires were not labeled with names and were collected in envelopes.

The questionnaire consisted of the following items:

(1) Personal characteristics: grade, sex, school avoidance or absenteeism.
(2) Game‐related behaviors. (a) Game playing time on weekdays and holidays: participants chose one of the following eight options: 0 min, 30 min or less, 1 h or less, 2 h or less, 3 h or less, 4 h or less, 6 h or less, or more than 6 h. (b) Average monthly expenditure on in‐game items: participants chose one of the following four options: “I've never paid for games,” “Less than $10 (exchange rate: 100 JPY = 1 USD),” “$10 to a few hundred dollars,” or “more.” (c) Starting age of gameplay: participants chose one of four options: “I don't play games,” “Before elementary school,” “Lower elementary school grades,” or “Upper elementary school grades.”
(3) Evaluation of gaming disorder. In the ICD‐11, gaming disorder is defined by three items: (a) impaired control over gaming (e.g., onset, frequency, intensity, duration, termination, context); (b) increasing priority given to gaming to the extent that gaming takes precedence over other interests and daily activities; and (c) continuation or escalation of gaming despite negative consequences. The pattern of gaming behaviors may be continuous or episodic and recurrent, resulting in marked distress or significant impairment of personal, family, social, educational, occupational, or other important areas of functioning.^28^ The ICD‐11 criteria were assessed using these four statements: (a) “I sometimes become obsessed with games and cannot stop even if I want to,” (b) “I prioritize games over my daily life,” (c) “I continue to play games despite problems in my daily life,” and (d) “My interpersonal relationships and daily life are impaired by my game playing.” Participants who answered “yes” to both (a) and (b) and “yes” to either (c) or (d) or both were defined as potentially at risk for gaming disorder (potential gaming disorder [PGD]).
(4) Young's Diagnostic Questionnaire for Internet Addiction (YDQ)^29^: The YDQ was used to assess participants' Internet addiction. Participants who answered “yes” to five or more of the eight questions that comprise the YDQ were rated as having a high YDQ score.
(5) Strengths and Difficulties Questionnaire (SDQ) self‐report^30^: The SDQ self‐report form was used to assess participants' psychological difficulties. The SDQ consists of five scales of five items each, which were scored using the standard SDQ scoring method.
(6) Impact of the COVID‐19 pandemic and forced changes in living conditions were assessed using the following three items: (a) increase in subjective anxiety in the COVID‐19 pandemic, (b) increase in game playing during the COVID‐19 pandemic, and (c) increase in the amount of money spent on in‐game items during the COVID‐19 pandemic. For each of these aspects, participants chose one of the following four options: decreased, unchanged, slightly increased, or markedly increased.

### Score evaluation and statistical analysis

All questionnaires were compiled manually, and obvious errors and multiple responses to a single item were treated as missing values. Missing values are summarized in Supporting Information Table 2. The responses of 4048 (80.2%) participants who fully answered the four questions related to the diagnosis of gaming disorder were used in subsequent analyses. These participants were divided into two groups: those who met the definition of PGD (282 participants) and those who did not meet the definition of PGD (3766 participants) (see Figure 1). Differences in the responses of these two groups to the respective questionnaire items were examined using the following tests. Fisher's exact test was used to assess school grade, sex, school avoidance or absenteeism, game playing time on weekdays and holidays, average amount of money spent per month on in‐game items, and starting age of gameplay; the Mann–Whitney *U* test was used for game playing time on weekdays and holidays, average amount of money spent per month on in‐game items, and starting age of gameplay; and Welch's *t*‐test was used for the YDQ and SDQ self‐report. For the three questions related to COVID‐19, participants who answered “slightly increased” or “markedly increased” were classified as the increased group, and the difference in the percentage of participants in the increased group compared with the PGD group and control group was examined using Fisher's exact test. The significance threshold was set to *p* < 0.05 for all tests.

### Simple and multiple logistic regression analysis

Logistic regression analysis was applied to predict the extent to which the factors examined in the questionnaire contributed to the propensity for PGD. We used IBM SPSS v. 28.0.1 (IBM Corp.), and samples with missing values were excluded from the analysis. A total of 2686 samples were analyzed (see Figure 1); these are summarized in Supporting Information Table 3. The dependent variable was PGD, and the following independent variables were selected: school grade, sex, YDQ, five subscales of the SDQ‐self report, school avoidance or absenteeism, time spent playing games on weekdays and holidays, money spent on games, starting age of gameplay, anxiety related to COVID‐19, time spent on games during the COVID‐19 pandemic, and money spent on games during the COVID‐19 pandemic.

A correlation matrix was created to verify the correlation between the independent variables, and we confirmed that there was no strong correlation between the independent variables (*r* > 0.80; see Supporting Information Table 4). First, simple logistic regression analysis was performed using the dependent variable and each independent variable to determine the regression coefficient, odds ratio (OR), and *p* value. Then, multiple logistic regression analysis was conducted for all independent variables. We used the omnibus test for the model coefficients and the Hosmer–Lemeshow test to check the goodness of fit of the logistic regression model. Significance levels were set to *p* < 0.05.

## RESULTS

### Game‐related behaviors

**Figure 2:**
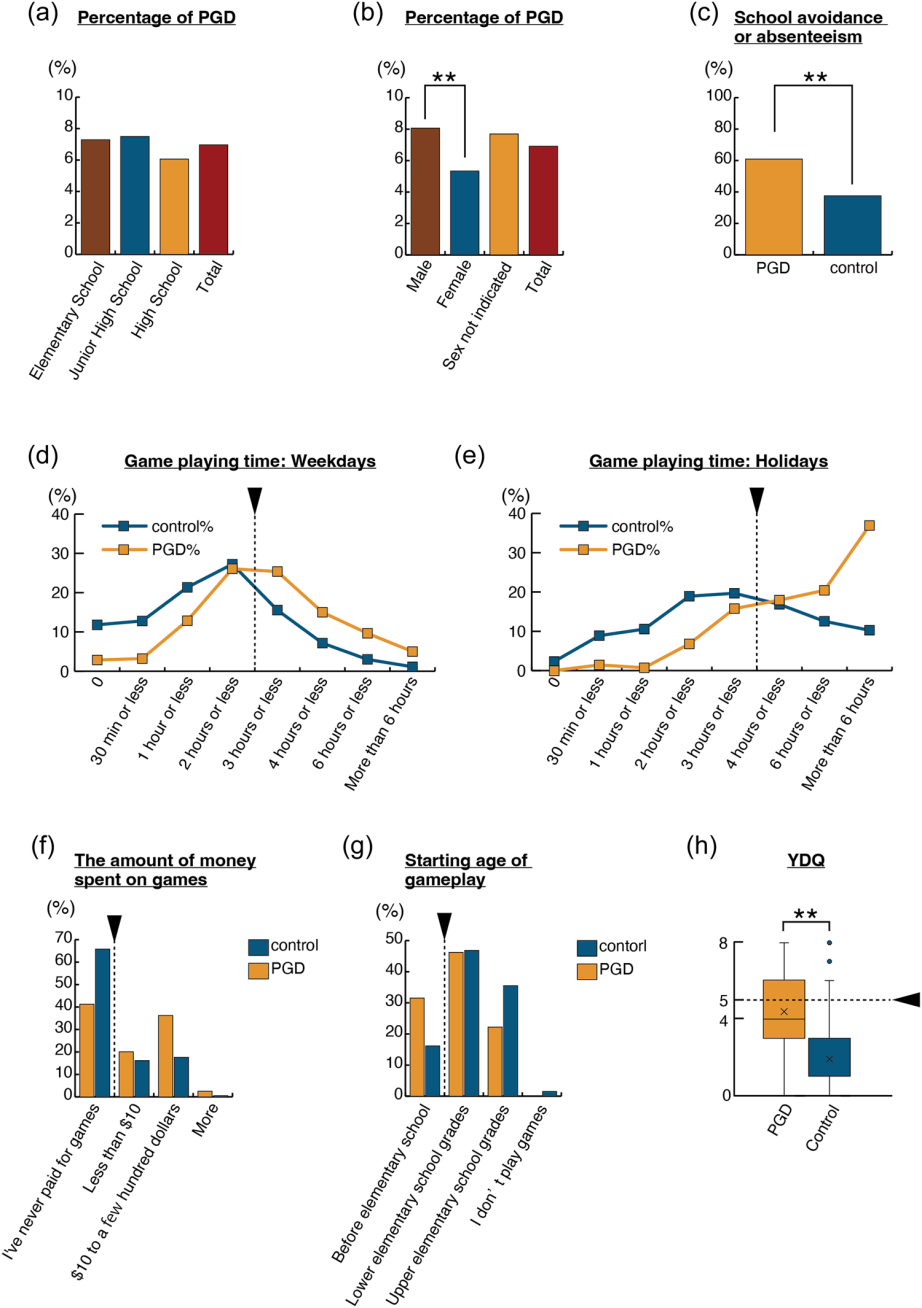
Questionnaire results on game‐related behaviors in elementary, junior high, and high school students during the COVID‐19 pandemic. (a) In total, 7.3% of elementary school students, 7.5% of junior high school students, 6.1% of high school students, and 7.0% of all participants met the criteria for PGD. (b) In total, 8.1% of male participants, 5.3% of female participants, and 7.7% of participants who chose not to indicate their sex met the criteria for PGD. The results for male and female students were significantly different. (c) In total, 60.9% of PGD participants and 37.5% of the control group reported school avoidance or absenteeism. Fisher's exact test showed that the difference between the two groups was significant. (d) Weekday game playing time for PGD and control groups. The number of participants who reported “2 h or less” of game playing time was lower in the PGD group than in the control group. Conversely, the number of participants who reported more than “3 h or less” of game playing time was higher in the PGD group than in the control group. (e) Holiday game playing time for PGD and control groups. The number of participants who reported “3 h or less” of game playing time was lower in the PGD group than in the control group. Conversely, the number of participants who reported more than “4 h or less” of game playing time was higher in the PGD group than in the control group. (f) Amount of money spent on games per month. (g) Starting age of gameplay. (h) YDQ score of PGD and control group participants: the difference between the means scores was significant. *p < 0.05, **p < 0.01. PGD, potential gaming disorder; YDG, Young's Diagnostic Questionnaire for Internet Addiction.

The highest percentage of students who met the criteria for PGD at 7.5% were in junior high school, followed by elementary school with 7.3% and high school with 6.1%. The differences between each school group were evaluated using Fisher's exact test and were not statistically significant (elementary school and junior high school, *p* = 0.884, OR = 0.97; junior high school and high school, *p* = 0.157, OR = 1.26; high school and elementary school, *p* = 0.224, OR = 0.82. The significance threshold was set to 0.017 in accordance with the Bonferroni correction) (see Figure 2a and Supporting Information Table 5). In terms of sex, male students (8.1%) accounted for the highest percentage of PGD participants, followed by the group that did not indicate their sex (7.7%) and female students (5.3%). The differences between each sex group were evaluated using Fisher's exact test, and the difference between male and female students was found to be statistically significant (males and females, *p* = 0.001, OR = 1.56; females and group with sex not indicated, *p* = 0.313, OR = 0.68; group with sex not indicated and males, *p* = 1.000, OR = 0.95; the significance threshold was set to 0.017 in accordance with the Bonferroni correction) (see Figure 2b and Supporting Information Table 6).

In the PGD group, 60.9% of participants reported that they experienced school avoidance or absenteeism. In contrast, 37.5% of the control group experienced school avoidance or absenteeism. The difference between the groups was significant (Fisher's exact test *p* < 0.0001; OR = 2.59) (see Figure 2c and Supporting Information Table 7).

The most common response regarding time spent playing games on weekdays was “2 h or less” in both the PGD and control groups. However, more participants reported longer game playing time in the PGD group than in the control group, and the difference between the groups based on the Mann–Whitney U test was significant (*p* < 0.0001). Fisher's exact test showed significant differences between groups of participants who indicated that their game playing time was longer than “3 h or less” (Fisher's exact test *p* < 0.0001, OR = 3.33) (see Figure 2d and Supporting Information Table 8). In contrast, the most common response to game playing time on holidays was “more than 6 h” in the PGD group but “3 h or less” in the control group. The difference between the two groups based on the Mann–Whitney *U* test was significant (*p* < 0.0001). Fisher's exact test showed significant differences between groups of participants who indicated that their game playing time was longer than “4 h or less” (Fisher's exact test *p* < 0.0001, OR = 4.63) (see Figure 2e and Supporting Information Table 8).

The percentage of participants who said they never paid for games was 48% in the PGD group and 69% in the control group. In contrast, a higher percentage of participants in the PGD group reported paying $10 to several hundred dollars or paying more than that ($10 to several hundred dollars: PGD group = 36.2%, control group = 17.6%; more: PGD group = 2.5%, control group = 0.48%). The difference between the two groups based on the Mann–Whitney U test was significant (*p* < 0.0001). Fisher's exact test showed significant differences among participants who reported that they never paid for games (Fisher's exact test *p* < 0.0001, OR = 0.36) (see Figure 2f and Supporting Information Table 9).

More participants in the PGD group started playing games before entering elementary school (PGD group = 31.5%, control group = 16.2%). However, more participants in the control group started playing games in the upper grades of elementary school (PGD group = 22.2%, control group = 35.5%). The difference between the two groups based on the Mann–Whitney U test was significant (*p* < 0.0001). Fisher's exact test revealed significant differences among participants who reported starting game playing before elementary school (Fisher's exact test *p* < 0.0001, OR = 2.14) (see Figure 2g and Supporting Information Table 10).

Next, the YDQ was used to investigate the participants' tendency toward Internet dependency. Participants with a score of 5 or higher were rated as high risk for Internet dependence. Of the participants who answered all the questions on the YDQ, 49.4% of the PGD group and 8.7% of the control group were in the high‐risk group. The mean YDQ score in the PGD group was 4.407 (standard deviation [SD] = 1.834), in contrast with 1.888 (SD = 1.706) for the control group. The difference between the means of the two groups was statistically significant (Welch's *t*‐test: *t*(291) = 21.163, *p* < 0.0001) (see Figure 2h).

### Impact of the COVID‐19 pandemic on game‐related behavior

**Figure 3:**
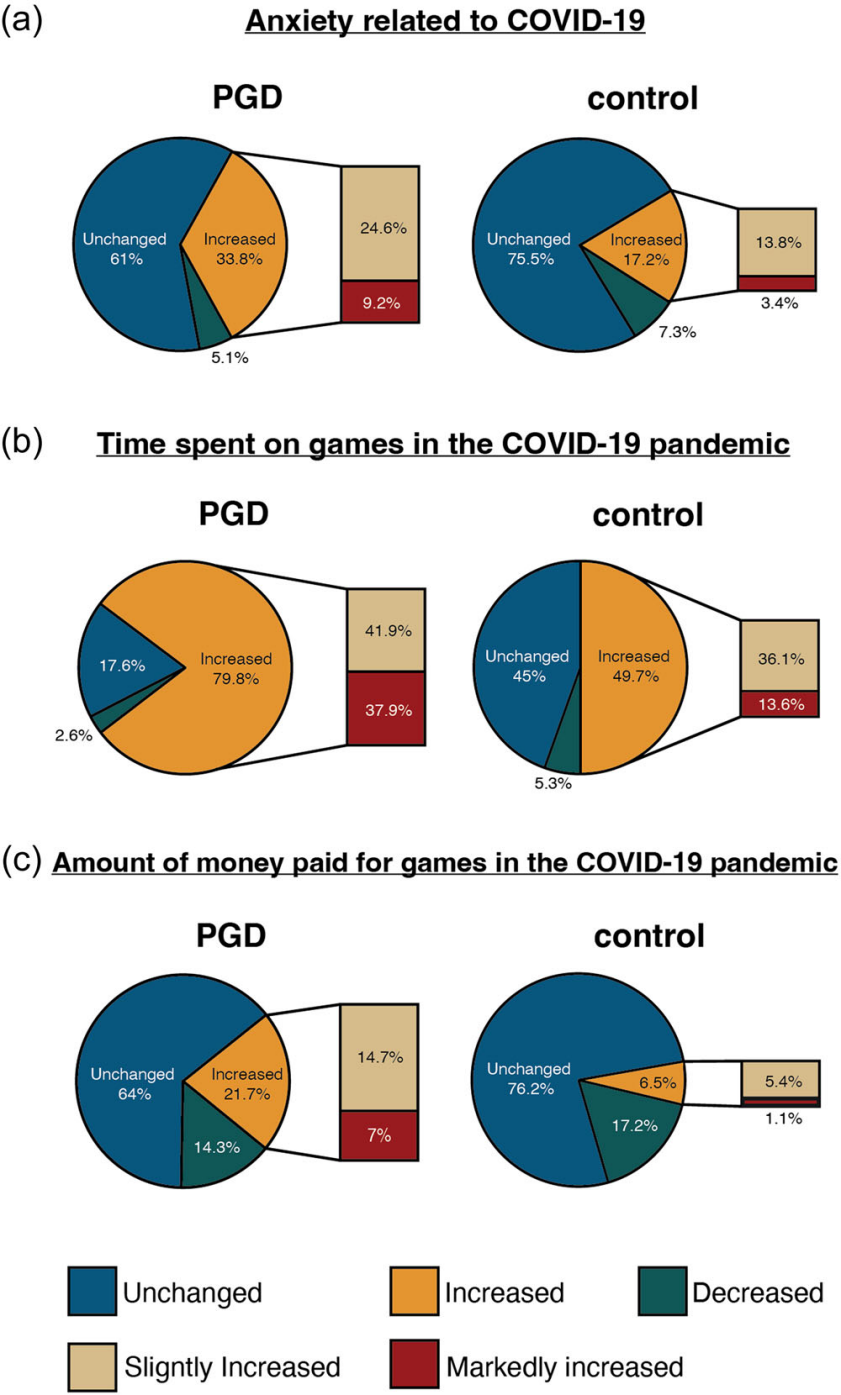
Impact of the COVID‐19 pandemic on game‐related behavior in children and adolescents. (a) In total, 33.8% of the PGD group reported increased anxiety (slightly increased = 24.6%, markedly increased = 9.2%) and 17.2% of the control group reported increased anxiety (slightly increased = 13.8%, markedly increased = 3.4%). (b) In total, 79.8% of the PGD group reported that their playing time increased (slightly increased = 41.9%, markedly increased = 37.9%) compared with 49.7% of the control group (slightly increased = 36.1%, markedly increased = 13.6%). (c) In total, 21.7% of the PGD group reported that the amount of money they spent on games increased (slightly increased = 14.7%, markedly increased = 7%) compared with 6.5% of the control group (slightly increased = 5.4%, markedly increased = 1.1%). PGD, potential gaming disorder.

We investigated anxiety caused by the COVID‐19 pandemic and changes in game‐related behavior. Of the participants who answered all three questions on COVID‐19, 33.8% of participants in the PGD group reported increased anxiety (slightly increased = 24.6%, markedly increased = 9.2%) and 17.2% of control participants reported increased anxiety (slightly increased = 13.8%, markedly increased = 3.4%) (see Figure 3a and Supporting Information Table 11). In total, 79.8% of participants in the PGD group reported that their playing time had increased (slightly increased = 41.9%, markedly increased = 37.9%) and 49.7% of participants in the control group reported increased playing time (slightly increased = 36.1%, markedly increased = 13.6%) (see Figure 3b and Supporting Information Table 11). In terms of money spent on games, 21.7% of participants in the PGD group said their spending had increased (slightly increased = 14.7%, markedly increased = 7%) and 6.5% of participants in the control group reported increased spending (slightly increased = 5.4%, markedly increased = 1.1%) (see Figure 3c and Supporting Information Table 11).

For all three COVID‐19‐related questions, a significant difference was noted between the PGD and control groups in the percentage of respondents who reported an increase in anxiety and game‐related behaviors during the pandemic (Fisher's exact test: anxiety related to COVID‐19 *P* < 0.0001, OR = 2.46; time spent on games during the COVID‐19 pandemic *P* < 0.0001, OR = 3.99; money spent on games during the COVID‐19 pandemic *P* < 0.0001, OR = 3.95).

### Psychological support needs of participants based on the SDQ self‐report

Of the 4048 participants who fully answered the questions related to the diagnosis of gaming disorder, 3749 participants (PGD group, *n* = 262; control group, *n* = 3487) fully answered all questions on the SDQ self‐report. We measured participants' scores on five subscales of the SDQ (emotional symptoms, conduct problems, hyperactivity/inattention, peer problems, and prosocial behavior) and the Total Difficulties Score (TDS, the sum of the four subscales except prosocial behavior) to determine their need for support. The mean score of the PGD group was higher than that of the control group in four of the subscales (emotional symptoms, conduct problems, hyperactivity/inattention, and peer problems) and the TDS. Additionally, the PGD group scored lower than the control group in prosocial behavior because only prosocial behavior is reverse‐scored. For all subscales and the TDS, the difference in mean scores between the two groups was statistically significant, suggesting the need for psychological support in the PGD group (Welch's *t*‐test: emotional symptoms, *t*(297) = 7.713, *p* < 0.0001; conduct problems, *t*(289) = 8.726, *p* < 0.0001; hyperactivity/inattention, *t*(296) = 9.492, *p* < 0.0001; peer problems, *t*(292) = 6.346, *p* < 0.0001; prosocial behavior, *t*(287) = 3.253, *p* = 0.001; TDS, *t*(298) = 12.121, *p* < 0.0001) (see Table 1).

**Table 1:** Scores on Strengths and Difficulties Questionnaire (SDQ) self‐report

SDQ self‐report	PGD (*n* = 262)	Control (*n* = 3487)	*t*‐test
	Mean (SD)	Mean (SD)	*p* value
Emotional symptoms	4.592 (2.474)	3.367 (2.338)	<0.0001
Conduct problems	3.630 (2.020)	2.505 (1.713)	<0.0001
Hyperactivity/inattention	5.126 (2.406)	3.672 (2.266)	<0.0001
Peer problems	3.695 (1.955)	2.899 (1.733)	<0.0001
Prosocial behavior	5.305 (1.940)	5.713 (1.581)	0.001
Total difficulties score	17.042 (5.927)	12.443 (5.730)	<0.0001

Abbreviation: PGD, potential gaming disorder.

### Predicted probabilities of PGD using multiple logistic regression

In simple logistic regression analysis, the *p* values of all independent variables were <0.05 and were statistically significant. Of these, the independent variable with the highest OR was the YDQ (OR = 9.59, 95% CI = 7.08–12.99), followed by holiday gaming time (OR = 5.74, 95% CI = 3.60–9.16) and money spent on gaming in the COVID‐19 pandemic (OR = 3.81, 95% CI = 2.70–5.38) (see Table 2).

**Table 2:** Summary of logistic regression analysis

Explanatory variables	Simple logistic regression analysis			Multiple logistic regression analysis		
	Regression coefficient	OR (95% CI)	*p* value	Regression coefficient	OR (95% CI)	*p* value
School grade	−0.067	0.94 (0.88–0.99)	0.029*	−0.083	0.92 (0.86–0.91)	0.028*
Male sex	0.351	1.42 (1.05–1.92)	0.022*	0.002	1.00 (0.68–1.48)	0.991
School avoidance or absenteeism	0.861	2.37 (1.78–3.15)	<0.001**	0.379	1.46 (1.05–2.03)	0.024*
Younger age at start of playing games	0.492	1.64 (1.35–1.99)	<0.001**	0.271	1.31 (1.05–1.63)	0.015*
YDQ	2.261	9.59 (7.08–12.99)	<0.001**	1.835	6.27 (4.46–8.81)	<0.001**
*SDQ self‐report*						
Emotional symptoms	0.654	1.92 (1.54–2.41)	<0.001**	0.212	1.24 (0.92–1.66)	0.159
Conduct problems	0.791	2.21 (1.75–2.78)	<0.001**	0.025	1.03 (0.76–1.38)	0.871
Hyperactivity/inattention	0.845	2.33 (1.86–2.92)	<0.001**	0.462	1.59 (1.19–2.03)	0.001**
Peer problems	0.573	1.73 (1.40–2.24)	<0.001**	0.297	1.35 (1.02–1.78)	0.036*
Prosocial behavior	0.337	1.40 (1.01–1.95)	0.044*	−0.012	0.99 (0.67–1.45)	0.949
*Game‐related behaviors*						
Time spent on games on weekdays	1.268	3.55 (2.48–5.09)	<0.001**	0.274	1.32 (0.83–2.10)	0.250
Time spent on games on holidays	1.747	5.74 (3.60–9.16)	<0.001**	0.983	2.67 (1.48–4.84)	0.001**
Money spent on games	0.531	1.70 (1.46–1.98)	<0.001**	0.277	1.32 (1.08–1.61)	0.006**
*COVID‐19*						
Anxiety related to COVID‐19	0.959	2.61 (1.93–3.52)	<0.001**	0.407	1.50 (1.06–2.14)	0.024*
Time spent on games in the COVID‐19 pandemic	1.247	3.48 (2.40–5.05)	<0.001**	0.144	1.16 (0.73–1.84)	0.544
Money paid for games in the COVID‐19 pandemic	1.338	3.81 (2.70–5.38)	<0.001**	0.633	1.88 (1.28–2.77)	0.001**

Abbreviations: CI, confidence interval; OR, odds ratio; SDQ, Strengths and Difficulties Questionnaire; YDQ, Young's Diagnostic Questionnaire for Internet Addiction.

* *p* < .005

** *p* < 0.01.

A correlation matrix was created before performing multiple logistic regression analysis. Here, the independent variables with a significant probability (<0.05) were school grade, school avoidance or absenteeism, younger age at start of game playing, YDQ, SDQ (hyperactivity/inattention and peer problems), time spent on games on holidays, money spent on games, anxiety related to COVID‐19, and money spent on games in the COVID‐19 pandemic. The independent variable with the highest OR was the YDQ (OR = 6.27, 95% CI = 4.46–8.81), followed by holiday gaming time (OR = 2.67, 95% CI = 1.48–4.84) and money spent on gaming in the COVID‐19 pandemic (OR = 1.88, 95% CI = 1.28–2.77) (see Table 2). The omnibus test of the model coefficients showed that the significance probability of the model was *p* < 0.001, confirming the significance of the regression equation. The significance probability of the Hosmer–Lemeshow test was 26.9%, indicating no apparent problem in the goodness of fit of the logistic regression model, and the positive discrimination rate was 92.5%.

## DISCUSSION

We conducted a cross‐sectional survey of game‐related behaviors among children and adolescents during the COVID‐19 pandemic. The PGD group was defined as participants who met three key components for the diagnosis of gaming disorder in the ICD‐11: loss of control over gaming, tendency to prioritize gaming over daily life, and continuing or escalating gaming despite negative consequences.

An estimated 7.0% of our sample were considered to have PGD. Reports on the prevalence of gaming disorder vary widely.^31^ Mihara et al. reported an IGD prevalence of 0.7–27.5%.^32^ Another review noted an IGD prevalence of 0.60–50.00% in children and adolescents,^33^ and slightly more conservative prevalence rates have also been reported.^34^, ^35^ The variability and heterogeneity of these estimated prevalence rates may reflect inaccurate diagnostic criteria and inconsistent assessment instruments across studies.^36^ Several useful scales have been developed for IGD as defined by the DSM‐5^37^, ^38^: the Bergen Social Media Addiction Scale (BSMAS),^39^ the Smartphone Application‐Based Addiction Scale (SABAS),^40^ and the Internet Gaming Disorder Scale‐Short Form (IGDS‐SF9).^41^, ^42^ Additionally, attempts are being made to develop rating scales for gaming disorder as defined by ICD‐11.^43^, ^44^, ^45^ Furthermore, variations and heterogeneity in the estimated prevalence rates may also be influenced by differences in regional and cultural backgrounds (e.g., prevalence may be higher in East Asian regions where gaming culture is more prevalent), methodological issues associated with the survey, and differences in participant demographics.^36^ Therefore, we emphasize the importance of standardizing research methods, such as assessment instruments, diagnostics, and surveys, across studies to accurately estimate the prevalence of IGD/gaming disorder in the future.

The PGD group in our study showed a variety of characteristic game‐related behaviors (e.g., extended game playing, especially on holidays, large sums of money spent on games, younger age at the start of game playing), a tendency toward Internet dependence, and a range of psychological support needs. A greater proportion of the PGD group than the control group experienced school avoidance or absenteeism. These results indicate that young people classified as having a gaming disorder based on the ICD‐11 diagnostic criteria may be psychologically and socially vulnerable and need support.

Our multiple logistic regression analysis suggested that factors associated with PGD included school grade, school avoidance or absenteeism, younger age at the start of game playing, YDQ, SDQ (hyperactivity/inattention and peer problems), time spent on games on holidays, and money spent on games. Many of these factors have been reported in previous studies, including school avoidance or absenteeism,^46^ younger age at the start of game playing,^47^ time spent on games,^48^, ^49^ and money spent on games.^48^ The YDQ score showed the highest OR, indicating a strong association between Internet dependence and PGD. This strong association between PGD and YDQ scores is not surprising, considering a report by Oka et al. showing that most of the risk factors are common in IGD and problematic Internet use.^50^ However, Oka et al. also reported that factors such as face‐to‐face communication time with family may differ between IGD and problematic Internet use. Differences in risk factors for gaming disorder and problematic Internet use should be examined in greater detail in the future.^50^

Nevertheless, the concordance of our results with those of previous studies reinforces the evidence that the factors identified here influence gaming dependence, and provides a rationale for early recognition of the potential risk of gaming disorder and therapeutic and preventive efforts to address gaming dependency.

Another important focus of this study was how the COVID‐19 pandemic affected students' mental health and game‐related behaviors. It is worth noting that participants in the PGD group tended to feel more anxiety about COVID‐19 than those in the control group. The former group spent more time playing games and spent more money on games during the COVID‐19 pandemic.

The OR for the amount of money paid for games during the pandemic was the third highest, after YDQ and time spent on games on holidays. This suggests that changes in game‐related behaviors attributable to COVID‐19 may have a relatively strong influence compared with other gaming disorder‐related factors.

Given the reports of strong associations between IGD and adverse psychological states such as anxiety and depression,^51^, ^52^, ^53^ and the accumulating evidence reporting an association between increased levels of psychological distress and Internet‐related behaviors during the COVID‐19 pandemic,^54^, ^55^ one interpretive model is that the COVID‐19 pandemic may have caused adverse psychological states in participants, exacerbating game‐related behaviors and the need for psychological support.

Another possible interpretive model is that participants who previously had psychological and social vulnerabilities may have been more strongly affected by the life changes caused by the pandemic. These models emphasize the importance of psychosocial support for children and adolescents during the COVID‐19 pandemic and the need to reduce the risk of gaming disorder. Thus, a more detailed investigation of game‐related behavioral changes associated with the COVID‐19 pandemic and identification of higher risks of gaming disorder‐related factors could be of great public health benefit.

Several limitations of this study should be noted. First, the determination of the PGD group relied on self‐reporting and no objective indicators were used. Therefore, it is unclear whether the game‐related difficulties and characteristic game‐related behaviors reported by participants represent a situation that should be evaluated as atypical.

The second limitation is that only limited information was collected on the neurodevelopmental vulnerability of the participants. In particular, there is growing evidence of the association between gaming disorders and neurodevelopmental disorders such as autism spectrum disorder and attention deficit hyperactivity disorder.^56^, ^57^ Therefore, further research is needed on the association between IGD/gaming disorder and neurodevelopmental vulnerabilities in the context of the COVID‐19 pandemic.

The third limitation is that we did not examine the duration of symptoms because this study was conducted very early in the COVID‐19 pandemic, shortly after the school closures in Japan. The ICD‐11 defines the persistence of game‐related symptoms for a period exceeding, for example, 12 months, as a requirement for the diagnosis of gaming disorder, and proposes shortening the duration of symptoms needed for the diagnosis when all three game‐related symptoms are present. There are several conflicting reports regarding the stability of symptoms in gaming disorder. Several studies have shown that excessive gaming tends to be relatively transient.^58^, ^59^ However, other studies have reported relatively long‐term (6 months or 2 years) symptom stability.^60^, ^61^ In addition, given the sequential changes in children's problematic Internet use and problematic gaming during the COVID‐19 outbreak and recovery periods,^62^ further research is needed on symptom stability in gaming disorder during the COVID‐19 pandemic.

Finally, the most important limitation of this study is its cross‐sectional design; no conclusions can be drawn about causal relationships among the investigated factors. The COVID‐19 pandemic is ongoing, and future longitudinal studies are needed to investigate causal relationships among the risk factors associated with gaming disorder to assess the impact of the pandemic on children and adolescents.

## CONCLUSION

In the present study, we investigated dependency on gaming among children and adolescents in the context of life changes brought about by the COVID‐19 pandemic. The results indicate that young people classified as having PGD exhibited characteristic game‐related behaviors and psychological and social vulnerability. Furthermore, COVID‐19‐derived anxiety and game‐related behavioral changes showed a relatively strong association with PGD. Our results indicate that young people classified as having a gaming disorder may be in need of special support, especially during critical phases of the COVID‐19 pandemic. This study contributes to the body of knowledge about factors associated with gaming disorder in children and adolescents, and may inform researchers and clinicians about ways to support young people to remain safe and healthy during the COVID‐19 pandemic.

## AUTHOR CONTRIBUTIONS

Naoki Yamamoto performed the experiments and data analysis. Naoki Yamamoto and Yoshiro Morimoto drafted the manuscript. Yoshiro Morimoto and Akira Imamura critically reviewed and revised the draft. All authors made substantial contributions to and drafted the manuscript, and approved the final version of the manuscript.

## CONFLICT OF INTEREST

The authors declare no conflicts of interest.

## ETHICS APPROVAL STATEMENT

All experimental procedures were approved by the Ethical Review Committee of Nagasaki University and the study followed the Ethical Guidelines for Medical and Health Research Involving Human Subjects.

## PATIENT CONSENT STATEMENT

All participants gave their voluntary consent to participate after receiving information about the study.

## Supporting information

Supporting information.

## Data Availability

All relevant data supporting the findings of this study are available within the article and its Supporting Information files, or from the corresponding author upon reasonable request.
